# Isolation and Biological Control of *Colletotrichum* sp. Causing Anthracnosis in *Theobroma cacao* L. in Chiapas, Mexico

**DOI:** 10.3390/jof11040312

**Published:** 2025-04-15

**Authors:** Nadia Denisse Rodríguez-Velázquez, Irene Gómez-de la Cruz, Guillermo López-Guillen, Belén Chávez-Ramírez, Paulina Estrada-de los Santos

**Affiliations:** 1 Laboratorio de Biotecnología Microbiana, Escuela Nacional de Ciencias Biológicas, Instituto Politécnico Nacional, Ciudad de Mexico 11340, CP, Mexico; plus_nadia@hotmail.com (N.D.R.-V.); gocri18@gmail.com (I.G.-d.l.C.); 2 Laboratorio de Fitopatología, Escuela Nacional de Ciencias Biológicas, Instituto Politécnico Nacional, Ciudad de Mexico 11340, CP, Mexico; 3 Campo Experimental Rosario Izapa, Instituto Nacional de Investigaciones Forestales, Agrícolas y Pecuarias, Tutxtla Chico 30875, CP, Mexico; lopez.guillermo@inifap.gob.mx

**Keywords:** anthracnose, biological control, *Colletotrichum*, *Paenibacillus*

## Abstract

Anthracnose is a phytosanitary issue caused by various species of *Colletotrichum*. This study aims to revise the presence of *Colletotrichum* in the south of Mexico (the Soconusco area in Chiapas) and assess the inhibitory capacity of *Paenibacillus* sp. NMA1017 against *Colletotrichum* in in vitro and field experiments. The study involved sampling pods with anthracnose from 17 sites in the Soconusco area, Chiapas, Mexico. The incidence of the disease ranged from 0.6 to 11.63%. A total of 142 isolates exhibiting the morphological characteristics of the *Colletotrichum* genus were obtained. Fifty selected isolates were identified using the ITS region and were classified as *Colletotrichum gloeosporioides* with 99% similarity. The concatenation of morphological and physiological characteristics resulted in nine main clusters. The in vitro test showed that *Paenibacillus* sp. NMA1017 inhibited the fungal growth of selected strains by 30–50%. The field experiments included three commercial biocontrol agents, *Paenibacillus* sp. NMA1017, and a water control. The incidence of anthracnose (control with water) ranged from 32 to 65%, while the commercial biocontrol agents and *Paenibacillus* showed an incidence range of 12 to 20%. These findings support the use of *Paenibacillus* sp. NMA1017 as a biocontrol agent for cacao anthracnose.

## 1. Introduction

Anthracnosis is a phytosanitary problem caused by different *Colletotrichum* species [1]. This ubiquitous pathogen affects mono- and dicotyledonous plants, mainly in tropical and subtropical regions. The main tropical fruits highly susceptible to anthracnose are mango, papaya, banana, avocado, and guava, with the post-harvest stage being the most affected, altering the marketability [2]. *Colletotrichum* species can be categorized as necrotrophic, hemibiotrophic, latent or quiescent, and endophytic. The life cycle begins when Colletotrichum spores in the environment or on plant surfaces are dispersed by wind, rain, or insects. These spores are deposited on the epidermis of the cacao pod. Under high humidity conditions and warm temperatures (typical conditions in cacao fields), the conidia germinate and begin to penetrate the host cells through the cuticle or natural openings (stomata) [3]. The hemibiotrophic phase is the most common phase [4], in which the pathogen remains in living plant tissues, absorbing nutrients but preventing the host cells from perishing [5] and then shifting to a necrotrophic lifestyle. During the primary infection, vesicles are formed without killing the cells. Then, the necrotrophic stage starts with a secondary infection in which hyphae invade and destroy adjacent cells [6,7]. The symptomatology of anthracnosis includes stem, leaves, and inflorescence cuticular and subcuticular lesions, which grow and destroy the tissue (necrosed tissue) [2,8]. In fruits, the lesions in the epidermis start as small, water-soaked circles that enlarge and become dark. They become soft and sink, growing up to 2 cm. Single lesions grow and merge, located only on the fruit surface, but can progress and invade the fruit pulp. In advanced stages, the fungus produces acervuli containing many conidia with a white to salmon color.

Within *Colletrotrichum,* different species cause anthracnose. However, *Colletrotrichum gloeosporioides* is the most frequently found [9]. The importance of this pathogen is due to its capacity to attack at distinct plant growth stages, including fruits in the postharvest phase, which is the source of low-quality fruit, thus affecting commercialization. Losses due to anthracnose are between 50 and 100% worldwide, depending on the host and abiotic factors [10]. In Mexico, there are reports of *Colletotrichum* attacking several hosts; some are avocados, which are harmed by at least seven species [11]. A complex of six *Colletotrichum* species [12] damages the coffee crop. Other hosts are mango, papaya, chili, and lemon [8,13]. In other countries, different species of *Colletotrichum* were reported in cacao [9]. The great economic importance of *Colletotrichum* as a ubiquitous phytopathogen has contributed significantly to the use of systemic fungicides, which provoke fungal resistance in the long term [14]. Therefore, it is recommended to use integrated management, including cultural practices and genetic, chemical, and biological control [13]. Biological control, or biocontrol, is defined as the use of microorganisms or metabolites synthesized by them to inhibit the growth of phytopathogenic fungi or bacteria. In *Colletotrichum* species, biological control has been partially explored in the field. However, some reports about the biocontrol of *Colletotrichum* include *Trichoderma viride*, *Bacillus licheniformis*, *Bacillus subtilis*, and *Rhodotorula diminuta* (now *Cystobasidium minutum*), which show efficiency in diminishing the phytopathogen incidence [8,13,15].

Previously, our research group isolated *Paenibacillus* sp. NMA1017 from *Opuntia ficus-indica* (L.) Mill. Barbary fig rhizosphere in Milpa Alta (Mexico City) [16]. This bacterium strongly inhibited several phytopathogens, including fungi, oomycetes, and bacteria. Moreover, the capacity of *Paenibacillus* sp. NMA1017, as a biocontrol agent of diseases, was tested in *Theobroma cacao*, mainly against *Phytophthora tropicalis* (black pod rot) and *Moniliophthora roreri* (moniliasis) [17,18] and *Hemileia vastatrix* in *Coffea arabica* [19]. The strain not only inhibited the phytopathogen growth or germination (*H. vastatrix*) in vitro experiments but also reduced the incidence of the diseases in the field using cacao and coffee crops.

The present study shows the features of *Colletotrichum* isolates obtained from cacao tree crops growing in the south of Mexico, the in vitro effect against *Colletotrichum* strains, and the impact of *Paenibacillus* sp. NMA1017 on cacao trees infected with anthracnose.

## 2. Materials and Methods

### 2.1. Survey Location

**Figure 1:**
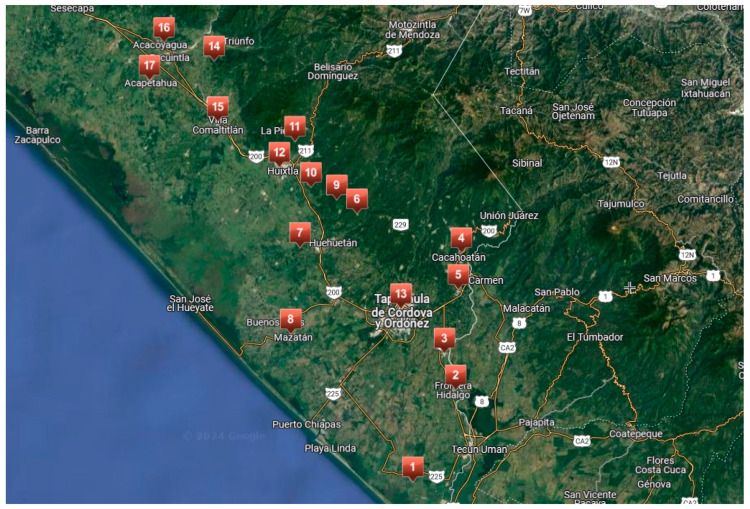
Map of the Soconusco area in Chiapas, Mexico, indicating the 17 sites where cacao pods with anthracnose were collected.

Seventeen sites in the Soconusco area in Chiapas (Tapachula in the southern to Acapetagua in the northern) were selected to collect diseased pods and leaves presenting anthracnose signs and symptoms (Table 1, Figure 1). Five points were selected in each site, and three diseased pods were collected from each point. The pod collection was performed in July 2021.

### 2.2. Incidence of Anthracnose

The incidence of anthracnose was determined in each site (from the 17) using the five-point sampling method during July 2021. At each point, five cacao trees were selected to count the total number of pods and the number of infected pods with characteristic symptoms where necrotic lesions appear with a sunken appearance of freckles, up to the mummification of the fruit with orange or pink sporulation. To calculate the incidence of the disease, the following formula was used: (%) = (number of pods infected with *Colletotrichum* number of pods evaluated) × 100 [20].

### 2.3. Isolation of Colletotrichum *sp.*

The cacao pods were washed with running water, and then a piece of the pericarpium was cut (5–10 mm^2^) from the border of the lesion in aseptic conditions. The inner cacao pod tissue fragments were obtained (2–3 mm) and placed in potato dextrose agar (PDA) Petri dishes. The plates were incubated at 30 °C for 72 h. After the incubation time, monosporic cultures were performed for each isolate. For that, a suspension of approximately 1 × 10^4^ conidia/mL was prepared for each fungal colony. An amount of 20 μL was placed in PDA, then plated with glass beads [21]. The plates were incubated as mentioned. Using a microscope (40×) (Carl Zeiss, Oberkochen, Germany), a single conidium was located, removed by cutting from the agar (2 mm^2^), and placed in a new PDA Petri dish. The plates were incubated as mentioned above. The isolates were kept on PDA slants at room temperature.

### 2.4. Molecular Identification

DNA extraction was performed according to Allers and Lichten [22]. The pathogen mycelia used for isolation were collected by gentle scraping with a sterile scalpel from a five-day-old culture grown on PDA. The isolated DNA was used to amplify the internally spaced sequence (ITS, ITS1-5.8S-ITS2) with primers ITS1/ITS4, resulting in 754 to 834 bp fragments [23]. PCR fragments were sequenced using the same primers at Macrogen Inc., (Seoul, Republic of Korea) (https://dna.macrogen.com/, accessed on 23 February 2025). The forward and reverse sequences were edited and assembled with ChromasPro v2.2.0 (Technelysium Pty Ltd., Brisbane, Australia). The consensus sequences were then compared with the *Colletotrichum* database. A sequence alignment was performed with MUSCLE (https://www.ebi.ac.uk/Tools/msa/muscle/, accessed on 23 February 2025). A phylogenetic tree was inferred using the maximum likelihood method with the PhyML 3.0 program under the GTR+I model [24] and 1000 bootstraps. The phylogenetic tree was visualized using the MEGA 11.0 program [25]. The identification was performed on 50 selected isolates, presenting different morphological and physiological characteristics.

### 2.5. Morphologic and Physiologic Fungal Characterization

Mycelial agar disks (5 mm diameter) were removed from the actively growing edge of five-day cultures of *Colletotrichum* sp. on PDA to observe colony and microscopic morphology. The plates were incubated at 28 °C. Colony features (color, appearance, and formation of concentric circles) were noted after five days. Microscopic analysis was performed using lactophenol preparations to describe conidia morphology and the presence of appressoria at a magnification of 400×.

The physiological characterization included determining the effect of temperature on mycelia growth (presence or absence) by inoculating the isolates on PDA using a 5 mm mycelium agar disk, which was then incubated for seven days at 28, 30, 37, and 45 °C [26]. Also, the growth on different media (PDA, cornmeal agar, and V8 agar) was performed by inoculating the isolates using a 5 mm mycelium agar disk and incubating at 28 °C for five days. Finally, to measure the growth rate, the isolates were inoculated on PDA and incubated at 28 °C for five days; the average growth rate was calculated by measuring the fungal growth over time [27,28].

### 2.6. Colletotrichum *sp.* Clustering Using Morphological and Physiological Characteristics

The data from the morphological experiments on colonial and microscopic morphology, growth in different media, and temperature growth rate were coded in a binary format of presence (one) and absence (zero), including the quantitative characters that were categorized into ranges due to their normal distribution. The coding was performed according to the guidelines of Crisci and Stuessy [29]. The algorithm used was the Unweighted Pair Group Method using Arithmetic averages (UPGMA), and the Jaccard similarity index was used to observe the similarity between communities using the program Paleontological Statistics (PAST) [28].

### 2.7. Pathogenicity Analysis

The experiment included nine isolates selected with different morphological and physiological characteristics. The pathogenicity analysis included pear fruits as a biological model. Given that cacao pods decay rapidly once detached from the tree, we proposed using pear fruits as an alternative model. Therefore, healthy pear fruits were surface sterilized with 3% sodium hypochlorite for 5 min and washed thrice with sterile distilled water. Using a needle, each pear was wounded, and a 5 mm agar plug containing mycelium from a five-day-old isolate was placed in the wound. An agar plug without microbial growth was placed in the wound for the control. The pear fruits were incubated in an aseptic plastic box at 28 °C. Wound development was observed daily for five days, and the phytopathogen was isolated. With this experiment, Koch’s postulates were fulfilled since they established the disease, the pathogen, disease development, and re-isolation of the pathogen. For each treatment, three biological replicates were prepared.

### 2.8. Efficacy of Paenibacillus *sp.* NMA1017 on Colletrotrichum *sp.* *In Vitro*

Strain NMA1017 was grown in potato dextrose broth (PDB) at 30 °C for 72 h in reciprocal shaking (150 rpm). Using serial dilutions, a culture suspension of strain NMA1017 was prepared at approximately 0.5 OD at 600 nm. In a Petri dish containing PDA, 5 μL of the bacterial suspension was placed in 8 points equidistant to the center of the dish. Then, a 5 mm diameter cylinder of fungal growth (5 days) was positioned in the Petri dish center. The fungi were inoculated in the center of a PDA plate as a control. The plates were incubated at 30 °C for five days. The formula from Ezziyyani et al. [30] was applied to obtain the fungal growth inhibition percentage.

Inhibition % = (Diameter of fungus control − Diameter of a fungus with bacteria) × 100/Diameter of fungus control.

### 2.9. Efficacy of Paenibacillus *sp.* NMA1017 on Colletrotrichum *sp.* in the Field

The assay was carried out in a cacao field called La Montaña, located in Tuxtla Chico, Cacahoatán, Chiapas, Mexico, with the geographical coordinates 14.982500, −92.153889. The field is 2 ha with 50% shade, four-year-old cacao plants, and planted with the criollo Carmelo variety. The first assay was performed between February and April 2021; the second was between June and August 2021. Before the experiment’s establishment, anthracnose’s incidence was assessed according to what was described in the anthracnose incidence section above. Fifteen pods were tagged per treatment. Each treatment was applied in different lines, and one line was left to separate each treatment (Figure S1). There were four replicates per treatment, meaning four lines per treatment, resulting in 60 tagged pods per treatment.

The applied treatments were (a) *Trichoderma* sp. CERI (b) SERENADE^®^ ASO (c) Fungifree^®^ AB containing *Bacillus subtilis* 1 × 10^9^ CFU at 200 g per 100 L of water. (d) *Paenibacillus* sp. NMA1017. (e) Water as control. Each treatment was prepared as follows: *Trichoderma* sp. CERI was isolated from cacao at Instituto Nacional de Investigaciones Forestales, Agrícolas y Pecuarias, Rosario Izapa, Chiapas, Mexico. The strain was grown in PDA plates for 72 h, then a spore suspension was prepared at approximately 1 × 10^5^ spores/mL. SERENADE^®^ ASO contains *Bacillus subtilis* QST 713 1.34%. The product was prepared by adding 10 g to 5 L of water. Fungifree^®^ AB contains *Bacillus subtilis* 1 × 10^9^ CFU at 200 g per 100 L of water. *Paenibacillus* sp. NMA1017 was isolated from *Opuntia ficus*-*indica* in Milpa Alta, Mexico City [16]. The strain was grown in PDA plates for three days at 30 °C. The growth from three PDA plates was resuspended in 10 mM MgSO_4_ 7H_2_O to obtain an optical density of approximately 0.5 (600 nm). Then, 5 mL was inoculated in 500 mL of PDB and incubated for three days at 30 °C (150 rpm). After the incubation, a culture of approximately 1 × 10^10^ CFU/mL was obtained and serially diluted to obtain approximately 1 × 10^8^ CFU/mL. The treatments were randomly applied in each line (1.5 L) every ten days for three months. After three months, the incidence of diseased pods was determined in tagged pods.

### 2.10. Trapping of Phytopathogen Spores

Traps covered with transparent Scotch tape were placed in the cacao trees to determine whether the phytopathogens were naturally present in the environment. The traps were checked at intervals of ten days before the application of treatments. Three plastic rectangles (12 × 1 cm) were positioned in the upper, middle, and lower parts five cacao trees following the five-point sampling method. Then, the tape was removed and placed on a slide with a drop of lactophenol cotton blue stain. The slides were observed at 40×.

### 2.11. Bacterial Effect on Mycelial Growth and Morphology

Fungal microcultures (in triplicate) were performed as follows: 250 μL of PDA (agar 7 g/L) were placed in a sterile excavated microscope slide (Global Scientific Inc., Nagpur, MA, USA). Then, in the PDA medium, an agar plug (about 1 cm^2^) inoculated with *Colletotrichum* sp. (5 days old) was placed in the center. On the top of the inoculated medium, 20 μL of PDB was added and covered with a sterile cover slide (control treatment). For the inoculation with *Paenibacillus* sp. NMA1017, the same procedure was carried out, but 20 μL of the bacterium culture in PDB was added. The microcultures were held in a humidity chamber for 3 days at 28 °C. The microscope slides were separately placed in a sterile glass Petri dish containing water:glycerol (2:1) to keep humidity. The interaction between fungi and bacteria was observed by optical and scanning electron microscopy (SEM). The optical observation was performed by scanning the entire surface (40×). For SEM, 5 to 10 samples were taken from the microculture and fixed with 2.5 glutaraldehyde in a 0.1 M phosphate buffer (pH 7.2) for 25 h at 4 °C. Then, alcohol dehydration was performed for 15 min using consecutively 10, 30, 50, 70, 96, and 100 ethanol solutions. Finally, the metallization of the dehydration samples was carried out and observed at 15 kV on a Joel JMS 6480 L V Computer (Joel Ltd., Peabody, MA, USA).

### 2.12. Data Analysis

The effectivity assay was repeated twice, and the data were analyzed using a randomized design. A normality analysis of the data was also included. Tukey’s comparison means were performed with GraphPad Prism v8.0.1.

## 3. Results

### 3.1. Anthracnose Incidence

The study of anthracnose incidence in cacao showed that the Frontera Hidalgo site had the highest value (11.63%), followed by Mazatán (4.28%), while the Tapachula site had the lowest incidence (0.6%) (Table 1).

### 3.2. Colletotrichum *sp.* Isolation

**Figure 2:**
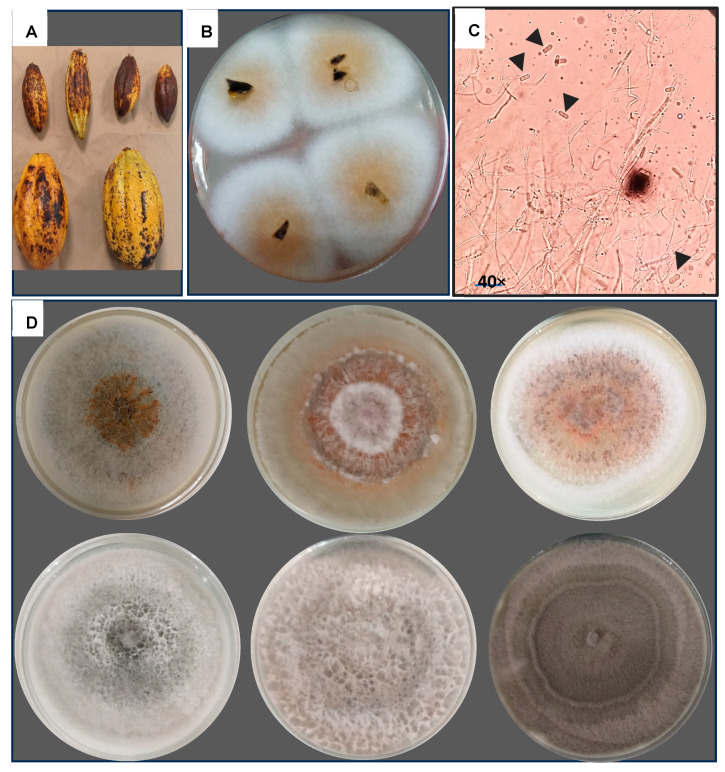
Isolation and identification of anthracnose in cacao pods. (**A**) Symptoms of anthracnose in cacao. (**B**) Isolation of *Colletotrichum* spp. from the pericarp of the pod. (**C**) Microscopic morphology corresponding to *Colletotrichum* spp., septate hyaline mycelium is observed with the production of ovoid hyaline conidia 100×; arrows point to conidia characteristic of the genus. (**D**) Different culture and morphological characteristics of *Colletotrichum* spp. isolated from cacao. Growth on potato dextrose agar after 5–7 days of incubation at 28 °C.

The cacao pods presented symptoms typical of anthracnose, such as semicircular lesions, some in the shape of concentric rings and others with freckles (Figure 2A). After the morphological and microscopic analysis, 142 isolates presented characteristics of *Colletotrichum* (Table 1, Figure 2B–D).

### 3.3. Molecular Identification

To corroborate that the *Colletotrichum* isolates belong to that genus, 50 were selected for molecular identification by sequencing the ITS region. The 50 isolates were selected according to phenotypic and physiological features. The comparison of the sequences in the NCBI database revealed 99% similarity to several ITS from *Colletotrichum gloeosporioides*. The phylogenetic analysis showed the association of the strains with different species of the *Colletotrichum gloeosporioides* complex, such as *C. aenigma*, *C. theobromicola*, *C. queenslandicum*, *C. asianum*, *C. tropicale*, *C. fructicola*, *C. siamense*, *C. musae* and *C. gloeosporiodes* (Figure S2); thus, the identification was only achieved at the genus level.

### 3.4. Morphological and Physiological Characterization of Colletrotrichum *sp.*

Once the isolates were corroborated as *Colletotrichum* sp., the strains were characterized. The colony morphology, regarding the color of the upper surface, showed a high proportion of gray (78%), while the reverse side predominantly exhibited black (62.7%). As for the colonial texture, the most frequent type was powdery (51.79%), followed by cottony (37.5%). Concentric ring formation was observed in 19.64% of the cases. On the other hand, most strains formed pycnidia (78.57%). The physiological characterization included the growth rate (mm/day) ranging from 6 to 19.9 mm per day, with 8 to 8.9 mm per day being the most common (33.93%). All strains grew on PDA, cornmeal, and V8 media. Growth at various temperatures revealed that all strains grew at 28 and 30 °C. However, 30% of the strains grew at 37 °C, and no growth was observed at 45 °C (Table S1). Lastly, in terms of microscopic morphology, all strains presented cylindrical, hyaline, non-septate conidia with broadly rounded ends, with very similar measurements ranging between 3.73 and 12.13 × 2.36–6.1 µm and 6.17–5.36 × 2.24–7.75 µm (Table S2).

### 3.5. Colletotrichum *sp.* Clustering Using Morphological and Physiological Characteristics

**Figure 3:**
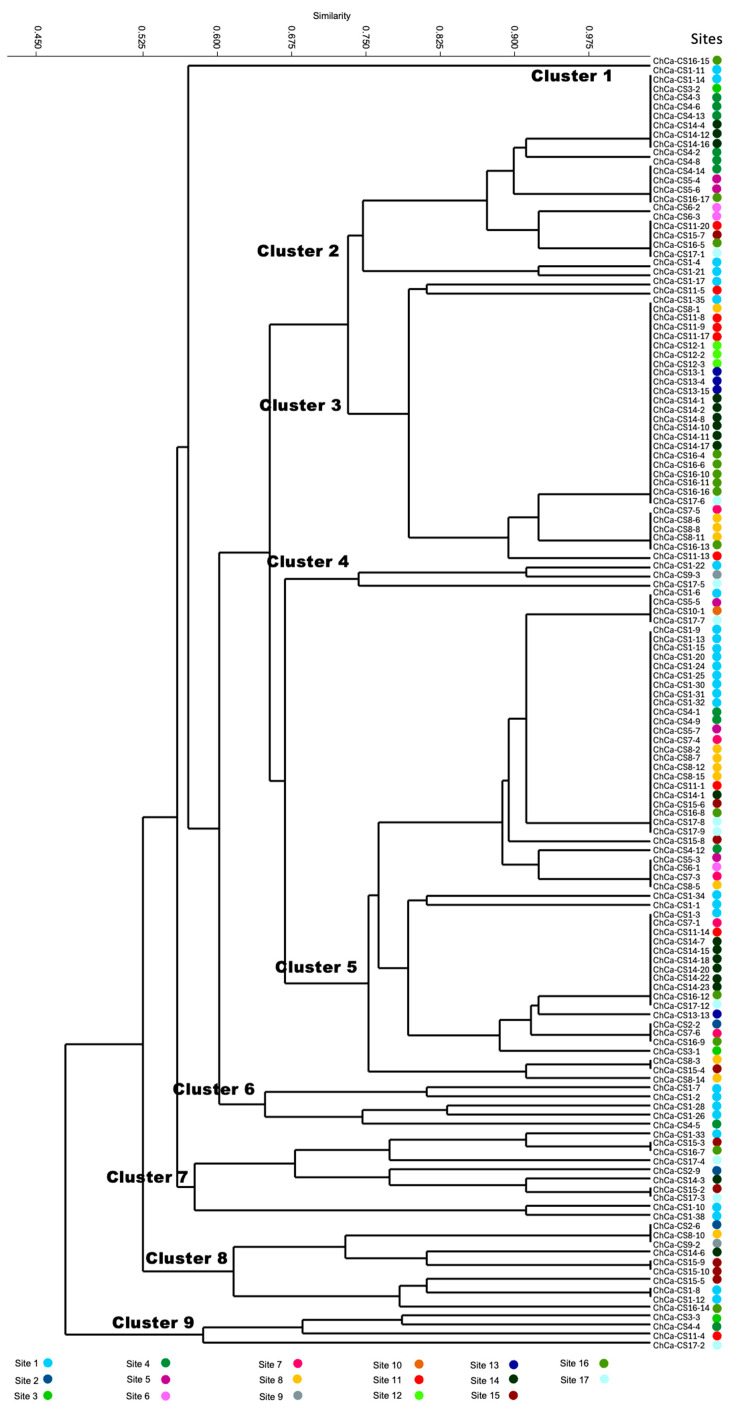
Dendrogram of morphological and physiological features of 142 *Colletotrichum* isolates. The analysis was performed using the Jaccard coefficient, and the dual-state data were calculated using the UPGMA clustering method.

The morphological and physiological characteristics clustering showed nine general clades within the dendrogram (Figure 3); some clades include strains from a few sites, but others from many sites, such as cluster 5, which contains *Colletotrichum* sp. obtained from sites 1, 4, 5, 8, 11, 13, 14, 15, 16, and 17, showing the high dispersion of one cluster.

### 3.6. Pathogenicity Test

**Figure 4:**
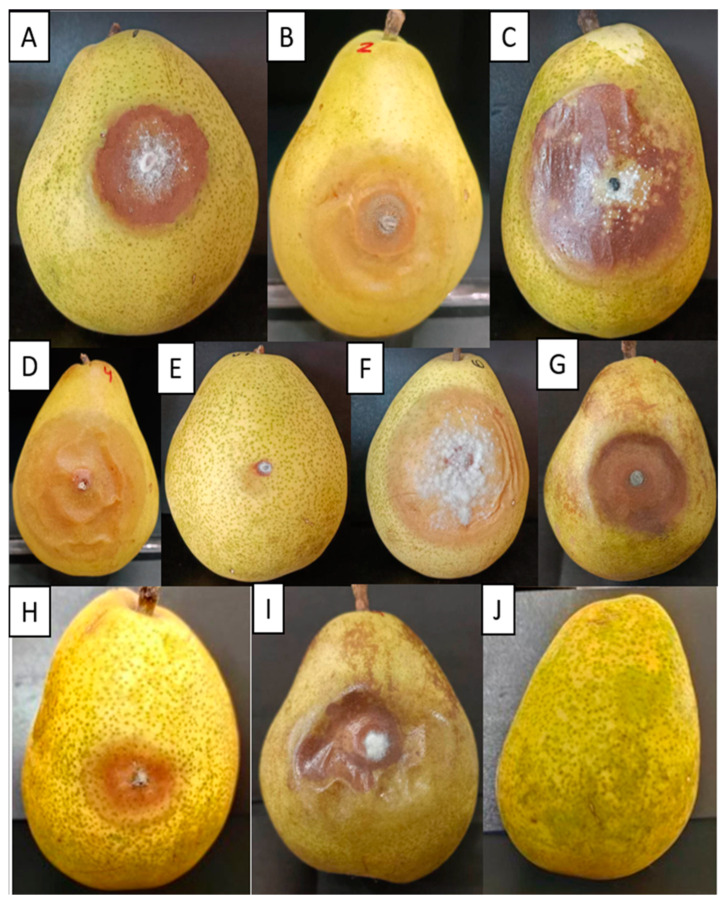
Pathogenicity test of selected isolates of the genus *Colletotrichum* spp. in pear fruits (variety D’Anjou USA). Incubated at 28 °C for 15 days. (**A**) ChCa-CS11-4; (**B**) ChCa-CS9-2; (**C**) ChCa-CS1-3; (**D**) ChCa-CS1-28; (**E**) ChCa-CS1-34; (**F**) ChCa-CS1-22; (**G**) ChCa-CS11-13; (**H**) ChCa-CS3-2; (**I**) ChCa-CS1-35; (**J**) Negative control.

The pathogenicity of *Colletotrichum* sp. strains was carried out using a pear fruit model. Nine strains were selected according to their morphological characteristics and the clustering analysis described in the previous section. The percentage of severity was 20% for strains ChCa-CS11-4 and ChCa-CS3-2, 40% for strain ChCa-CS9-2, 60% for strain ChCa-CS11-13, and 80% for strains ChCa-CS1-3, ChCa-CS1-28, ChCa-CS1-22, and ChCa-CS1-35 (Figure 4). In addition, some strains produced significantly larger lesions than others. Confirmation of pathogenicity was achieved by reisolating the strains from the inoculated pear fruit. The conidia colonies’ shape, size, and morphology were identical to the original strain inoculated.

### 3.7. Inhibition of Colletrotrichum *sp.* by Paenibacillus *sp.* NMA1017 *In Vitro*

In the present study, the in vitro antagonistic activity of *Paenibacillus* sp., NMA1017, was tested with the nine *Colletotrichum* strains previously selected for the pathogenicity test.

**Figure 5:**
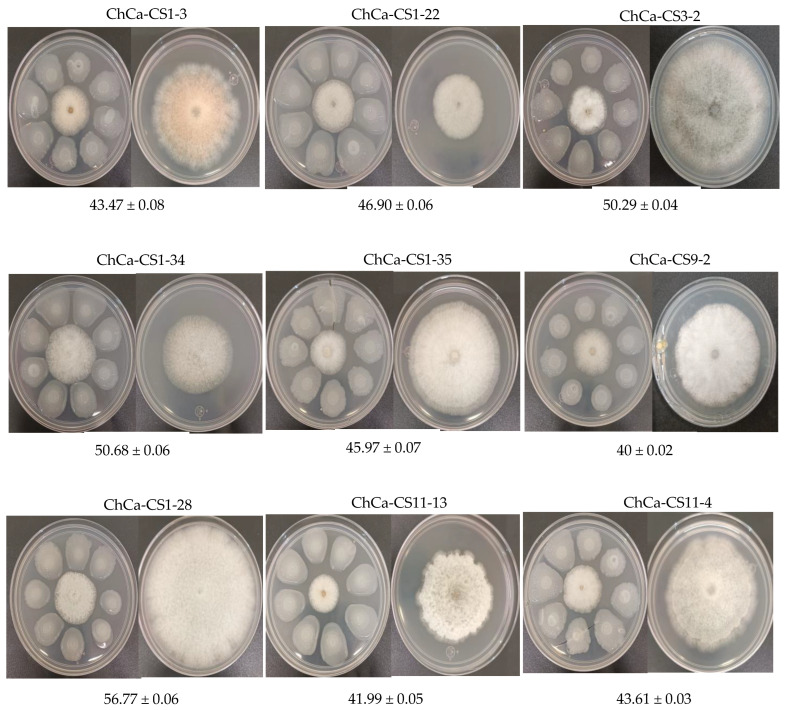
Inhibition of different *Colletotrichum* strains by *Paenibacillus* sp. NMA1017 in in vitro analysis. Below each test are the inhibition percentage values.

The inhibitory effect was observed with a significant clear zone of mycelial growth. Estimates showed that *Paenibacillus* sp. NMA1017 inhibited fungal growth from 30% to 50% (Figure 5). The percentage of inhibition was calculated using a one-way ANOVA test (*p* < 0.0001). Pairwise comparisons were performed using Tukey’s test, with a 95% confidence interval. The results showed that only the comparison of *Paenibacillus* sp. NMA 1017 vs. ChCa-CS1-3 and vs. ChCa-CS11-4 was not significant (*p* = 0.0001).

### 3.8. Field Bioassays with Cacao Trees

**Figure 6:**
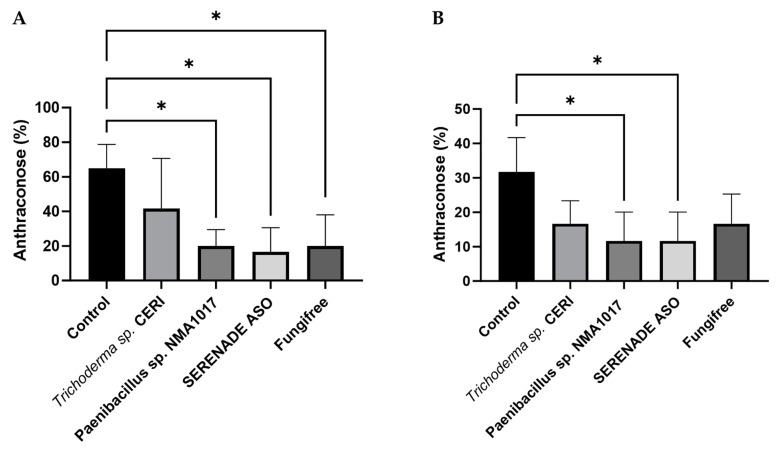
The average incidence of anthracnose on cacao pods after applying several biocontrol agents. (**A**) The first assay was carried out between February and April 2021. (**B**) The second assay was applied between June and August 2021. *, *p* > 0.05. The comparison was made with a one-way ANOVA test; multiple comparisons were made with Tukey’s test, assuming a value of *p* = 0.05 * as statistically significant.

The variance analysis in the first experiment points to statistical differences (*p* = 0.007) in the incidence of anthracnose in pods from each treatment. The mean comparison analysis test revealed a significant decrease in the incidence of anthracnose in pods treated with Fungifree AB (*p* = 0.022), Serenade ASO (*p* = 0.013), and *Paenibacillus* sp. NMA1017 (*p* = 0.022), regarding the control. The treatment with *Trichoderma* sp. CERI showed a decrease in anthracnose incidence. However, this was not significant (*p* = 0.396) (Figure 6A).

The variance analysis in the second experiment showed statistical differences (*p* = 0.0261) in the incidence of anthracnose with the treatments applied. The media analysis test indicates statistical differences in the incidence of anthracnose in pods treated with Serenade ASO (*p* = 0.031) and inoculated with *Paenibacillus* sp. NMA1017 (*p* = 0.031) compared to the control (Figure 6B). The treatment with *Trichoderma* sp. CER (*p* = 0.142) and Fungifree AB (*p* = 0.142) showed a decrease in the incidence of anthracnose, but the result was not significant.

**Figure 7:**
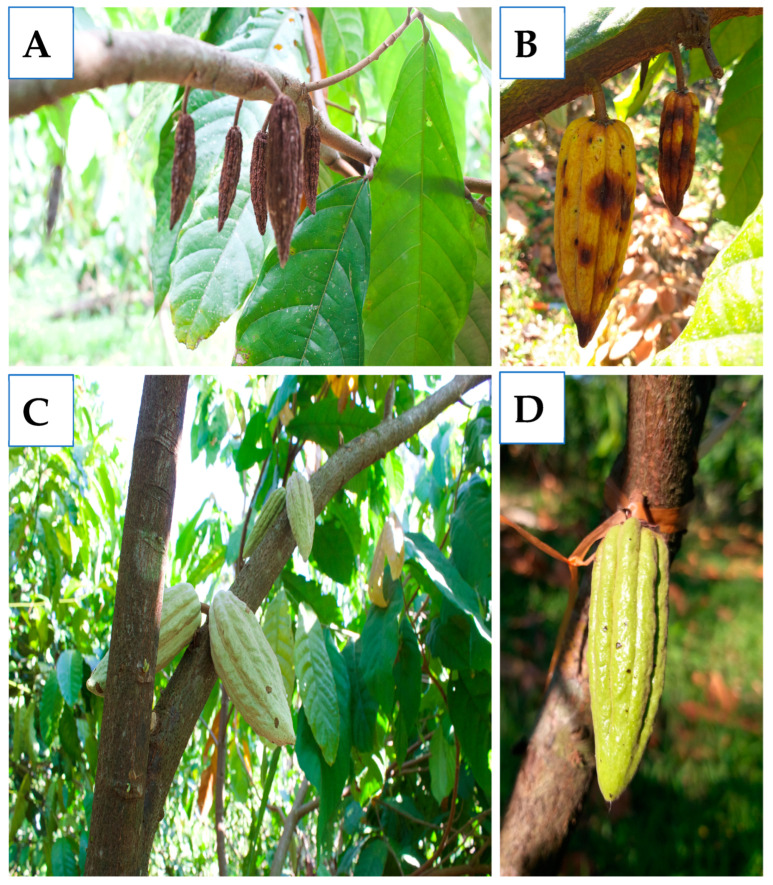
Efficacy of *Paenibacillus* sp. NMA1017 on anthracnose in cacao trees growing in Chiapas, Mexico. (**A**,**B**) Necrotized cacao pods by anthracnose (control pods). (**C**,**D**) Cacao pods treated with *Paenibacillus* sp. NMA1017.

The incidence of anthracnose in pods treated with water (control) was 65% for the first assay and 32% for the second. The incidence of anthracnose in pods treated with *Trichoderma* sp. and Fungifree was 17%, while in pods treated with Serenade ASO and *Paenibacillus* sp. NMA1017 was 12% in the first assay (Figure 7). In the second assay, the incidence of anthracnose in pods treated with *Paenibacillus* sp. NMA1017, SERENADE ASO, and Fungifree was 20, 20, and 17%, respectively.

### 3.9. Trapping of Phytopathogen Spores and Phytopathogen and Bacterial Isolation

The presence of *Colletotrichum* sp. spores in the cacao fields was monitored during the two efficacy bioassays. The analysis showed the presence of spores, which corroborated the inoculum to infect the cacao pods. Moreover, the phytopathogen and the biocontrol agent were isolated from the inoculated plants.

### 3.10. Bacterial Effect on Colletotrichum *sp.*

The evaluation of *Paenibacillus* sp. NMA1017 on *Colletotrichum* sp. hyphae using microcultures showed that the bacterium diminishes the number of conidia and hyphae elongation. The bacterium also affects the integrity of the fungal cell wall and membrane; in some cases, the hyphae were thinner (Figure S3).

## 4. Discussion

Anthracnose produced by *Colletotrichum* is a phytosanitary problem of global importance. *C. gloeosporioides* and *C. siamense* have been reported to affect leaves, flowers, and cacao pods, mainly in Venezuela [31]. In Mexico, research has focused on *C. gloeosporioides* and *C. trucatum*, which affect economically essential crops such as papaya and avocado [13]. In cacao, 3.7% of producers report anthracnose as a problem affecting the pod yield [18].

Studies on the genetic diversity of *Colletotrichum* in Mexico indicate that it is high. However, the impact and research on the disease in cacao fields are limited [28]. In 2015, the incidence of anthracnose was 3.2% in cacao fields in Mexico [13,32]. Currently, there are no new reports of anthracnose in Mexico. Therefore, this investigation focused on cacao production in the Soconusco area in Chiapas. The region comprises a surface of 4644.07 km^2^, from the border of Guatemala in the municipality of Frontera Hidalgo to the north in the municipality of Acacoyagua [33]. The incidence was variable but primarily low, with 0.6% in Tapachula and up to 11.63% in Frontera Hidalgo. Incidence studies of *Colletotrichum* in cacao in Mexico reveal essential insights into the epidemiology and management of this pathogen. Different studies indicated that *C. gloeosporioides* is a primary cause of foliar disease and pod rot in cacao, with infection rates increasing during the rainy season (June–November) due to favorable weather conditions [34]. This is certain because, during the first efficacy assay that was performed between the dry season in February and April, the incidence in control was 65%, while in the second, carried out between the beginning of the rainy season in June and August, it was 32%.

During the survey, 142 isolates were recovered in this study, presenting typical features of *Colletotrichum*, such as white-orange and gray-black color, with concentric rings and pycnidia formation. The morphological characteristics are a factor that may be related to the physiological and pathogenic status of the fungus, so it is essential to correlate these features [35]. Some authors have mentioned that the main differences among *Colletotrichum* strains are the growth and color, as it is the formation of acervuli on the plant tissue and features such as aspect, texture, and growth rate [9,36]. The powdery, velvety, and cottony aspects of the strains of *Colletotrichum* isolated in this study are similar to the ones presented by *C. truncatum* and *C. gloeosporioides* [9,37]. The microscope morphology showed typical hyaline ovoid conidia of the *Colletotrichum* genus [37].

Regarding the pathogenic characterization, different degrees of damage were found, corroborating that these isolates have a significant variability in virulence, giving rise to the fact that they can infect other fruits such as pears. The phenotypic features described in the present study helped to build a phenogram. In such a phenogram, similar strains from different isolation sites were located in the same clade, indicating a potential dispersion of the strains through rain, vectors, or manipulation of the cacao plants by people [38].

The host specificity of *Colletotrichum* spp. is challenging to establish, given that it has been demonstrated that the species have a broad host range; among the hosts documented are citric plants, seeds, and fruits like pear, apple, papaya, avocado, or cacao [39]. *Colletotrichum* can be found as a pathogen but also as a biotrophic fungus without causing any harm, which makes it hard to define the specificity of the species [38]. In this study, an analysis of the disease severity was performed using pear fruit as a model. Several lesions were dry, necrotic, profound, and had fungal growth beyond the inoculation point with sporulation, which helped to determine strain virulence. Moreover, it has been established that the severity of the disease in a pear fruit model correlates with experiments in cacao pods [40,41]. It has been mentioned that the pathogenicity can be variable depending on the strain analyzed [41,42], given different factors such as the quick evolution of the pathogenicity genes in *Colletotrichum* [43,44].

In Mexico, 82 *Colletotrichum* species have been reported causing disease in monocotyledonous plants [13]. Therefore, in this study, the biocontrol of anthracnose was examined. The biocontrol agent *Paenibacillus* sp. NMA1017 has been successfully applied to control black pod rot and frosty pod in Mexico’s cacao and coffee leaf rust [17,18]. The in vitro inhibition of *Colletotrichum* strains isolated in this study by *Paenibacillus* sp. NMA1017 was 40 to 56.77%, matching the results from Kim et al. [45], who showed that *Paenibacillus polymyxa* APEC136 inhibited *C. gloeosporioides* in vitro. The same strain used in in vivo experiments with apples showed an inhibition of 83.6% of *C. gloeosporioides* and 73% of *C. acutatum* [46].

The field evaluation of *Paenibacillus* sp. NMA1017 showed an anthracnose incidence inhibition of 63 to 69% in cacao pods. These results are substantially better than the ones reported by *Bacillus* sp., which diminished 40% of the incidence of anthracnose in cacao leaves [47]. The field evaluation of *Paenibacillus* sp. NMA1017 is an approach to evaluating the biocontrol effect of anthracnose in cacao in natural conditions, given that other studies in Mexico have been performed in controlled experiments due to challenging field conditions for the biocontrol agents to establish. However, some studies have shown the application and control by *Cystobasidium minutum* and *Bacillus subtilis* in mango plants, reducing anthracnose up to 86.7% in postharvest in Sinaloa, Mexico [48]. Among the commercial products evaluated in this study was Fungifree, based on *B. subtilis* 83, developed in Mexico to tackle the incidence and severity of anthracnose in mango, avocado, and papaya [49]. This product inhibited anthracnose in mango up to 71%, and in this study, the inhibitory effect on cacao was 47 to 69%. SERENADE ASO, based on *B. subtillis* QST713, is recommended against anthracnose [50]. The inhibitory effect of SERENADE ASO against anthracnose was 63 to 69%. These results showed that *Paenibacillus* sp. NMA1017 might be considered similar to commercial biocontrol agents.

Although the mechanism of protection from *Paenibacillus* sp. NMA1017 is currently under investigation in our laboratory, other authors suggest that different species, such as *P. polymyxa* TP3, reduce the incidence and severity of anthracnose in strawberries through the synthesis of antimicrobial peptides [51], volatile compounds [52], and production of enzymes like proteases and amylases [46]. Previously, we have determined that strain NMA1017 produces hydrolytic enzymes such as cellulose, xylanase, chitinase, and protease [15]. Moreover, the strain causes hyphae deformation in *Rhizoctonia solani* [29] and deformation and holes in *P. tropicalis* hyphae [16], which can be due to hydrolytic enzymes. The microcultures performed in the present study with *Paenbacillus* sp. NMA1017 vs. Colletotrichum sp. also showed hyphae deformation, suggesting a hydrolytic effect.

## 5. Conclusions

The present study shows a novel contribution to the biological control of *Colletotrichum* in cacao crops. The novelty lies in isolating different *Colletotrichum* strains directly from cacao crops in the Soconusco region of Chiapas, an area with rich biodiversity and unique conditions for cacao cultivation in Mexico. This is the first study to report the use of *Paenibacillus* sp. NMA1017, a bacterium isolated explicitly by our research group, as a biological control agent against *Colletotrichum* strains in this region. Additionally, our experimental approach includes both in vitro and field trials, allowing for a more comprehensive and realistic evaluation of the effectiveness of *Paenibacillus* sp. NMA1017 in controlling cacao anthracnose. This integrated approach, which combines the isolation of local *Colletotrichum* strains with a specific biological control, represents a significant advance in the sustainable management of fungal diseases in cacao.

This study suggests that *Paenibacillus* sp. NMA1017 has promising potential as a biological control agent against anthracnose in cacao trees, potentially reducing chemical fungicides and promoting integrated disease control.

## Figures and Tables

**Table 1:** Location sites in the Soconusco area, Chiapas, Mexico, where cacao pods infected with anthracnose were obtained, and number of isolates of *Colletotrichum* spp.

Site	County	Coordinates	Isolates	Incidence %
1	Suchiate	14°40′47.8 N 92°10′15.6 W	29	1.66
2	Frontera Hidalgo	14°45′07.8 N 92°11′27.6 W	3	11.63
3	Metapa	14°48′56.5 N 92°12′01.4 W	3	1.06
4	Cacahoatán	14°59.540′ N 092°11.166′ W	12	2.97
5	Tuxtla Chico	14°54.112′ N 092°11.942′ W	5	3.22
6	Huehuetán (Tepehuitz)	15°03.882′ N 092°20.078′ W	3	2.76
7	Huehuetán (Cantón Santa Elena)	15°00.797′ N 092°26.476′ W	5	1.49
8	Mazatán	14°49.651′ N 092°28.429′ W	12	4.28
9	Tuzantán (Cantón el Vado)	15°05.193 N 092°22.232′ W	2	3.55
10	Tuzantán (Cantón el Capulín)	15°06.625′ N 092°26.231′ W	1	1.77
11	Huixtla (Piedra Canoa)	15°10.608′ N 92°26.410′ W	9	2.24
12	Huixtla (La Unión)	15°03.686′ N 092°30.376′ W	3	1.49
13	Tapachula	14°52.565′ N 092°21.413′ W	4	0.6
14	Villa Comaltitilán (Monteflor)	14°52.555′ N 092°21.426′ W	18	3.02
15	Villa Comaltitlán (Los Cocos)	15°19.526′ N 092°34.261′ W	9	5.69
16	Acacoyagua (Ejido los Cocos)	15°23.628′ N 092°39.466′ W	14	3.41
17	Acacoyagua (27 de Mayo Amp. Madronal)	15°23.625′ N 092°39.467′ W	10	2.2

## Data Availability

The original contributions presented in this study are included in the article and Supplementary Material. Further inquiries can be directed to the corresponding authors.

## Supplementary Materials

The following supporting information can be downloaded at: https://www.mdpi.com/article/10.3390/jof11040312/s1, Figure S1: Location in Chiapas, Mexico (A) and experimental design (B) for applying *Paenibacillus* sp. NMA1017 to cacao trees for biocontrol of anthracnose. Each background color corresponds to different treatments. The white background color corresponds to a barrier between treatments; Figure S2: Phylogenetic analysis of *Colletotrichum* species using the ITS region sequence. The analysis used the maximum likelihood method and the GTR+I model with the program PhyML v 3.0. *Colletotrichum karstii* ICMP 113087 was used as an outgroup. In bold letters are indicated the strains analyzed in this study. The numbers in the tree correspond to the bootstrap analysis (1000). Bar, 0.0050 nucleotide substitutions per position; Figure S3: Effect of *Paenibacillus* sp. NMA1017 on *Colletricuhum* sp. in microcultures test. (A) optical observation of the control (40×), the image shows many conidia and the arrow indicates typical conidia. (B) optical observation of the effect of strain NMA1017 vs. *Colletotrichum* sp. (40×), the arrow points out a few conidia. (C) scanning electron microscopy of the effect of strain NMA1017 vs. *Colletotrichum* sp., the arrow specify damage to hypae. Table S1: Morphological and physiological features of *Colletotrichum* spp. isolated from diseased cacao pods in Chiapas, Mexico. Table S2: Size of representative strains of *Colletotrichum* spp.

## References

[1] Sharma M., Kulshrestha S. (2015). *Colletotrichum gloeosporioides*: An anthracnose causing pathogen of fruits and vegetables. Biosci. Biotechnol. Res. Asia.

[2] Zakaria L. (2021). Diversity of *Colletotrichum* species associated with anthracnose disease in tropical fruit crops, a review. Agriculture.

[3] da Silva L.L., Moreno H.L.A., Correia H.L.N., Santana M.F., de Queiroz M.V. (2020). *Colletotrichum*: Species complexes, lifestyle, and peculiarities of some sources of genetic variability. Appl. Microbiol. Biotechnol..

[4] Rodríguez-Velázquez N.D., Gómez-de la Cruz I., Chávez-Ramírez B., Estrada-de los Santos P., Kumar A., White J., Singh J. (2024). Biological control of diseases in *Theobroma cacao*. Plant and Soil Microbiome: Biocontrol Agents for Improved Agriculture.

[5] Mendgen K., Hahn M. (2002). Plant infection and the establishment of fungal biotrophy. Trends Plant Sci..

[6] Perfect S.E., Hughes H.B., O’Connell R.J., Green J.R. (1999). *Colletotrichum*: A model genus for studies on pathology and fungal-plant interactions. Fungal Genet. Biol..

[7] Barimani M., Pethybridge S.J., Vaghefi N., Hay F.S., Taylor P.W.J. (2013). A new anthracnose disease of pyrethrum caused by *Colletotrichum tanaceti* sp. nov. Plant Pathol..

[8] Jeschke P., Starikov E.B. (2022). Agricultural Biocatalysis: Biological and Chemical Applications.

[9] Rojas E.I., Rehner S.A., Samuels G.J., Van Bael S.A., Herre E.A., Cannon P., Chen R., Pang J., Wang R., Zhang Y. (2010). *Colletotrichum gloeosporioides* s.l. associated with Theobroma cacao and other plants in Panama: Multilocus phylogenies distinguish host-associated pathogens from asymptomatic endophytes. Mycologia.

[10] Siddiqui Y., Ali A., Bautista-Baños S. (2014). Colletotrichum gloeosporioides (Anthracnose). Postharvest Decay.

[11] Fuentes-Aragón D., Silva-Rojas H.V., Guarnaccia V., Mora-Aguilera J.A., Aranda-Ocampo S., Bautista-Martínez N., Téliz-Ortíz D. (2020). *Colletotrichum* species causing anthracnose on avocado fruit in Mexico: Status. Plant Pathol..

[12] Cristóbal-Martínez A.L., de Jesús Yáñez-Morales M., Solano-Vidal R., Segura-León O., Hernández-Anguiano A.M. (2017). Diversity of *Colletotrichum* species in coffee (*Coffea arabica*) plantations in Mexico. Eur. J. Plant Pathol..

[13] Rojo-Báez I., Álvarez-Rodríguez B., García-Estrada R.S., León-Félix J., Sañudo-Barajas A., Allende-Molar R. (2017). Situación actual de *Colletotrichum* spp. en México: Taxonomía, caracterización, patogénesis y control. Rev. Mex. Fitopatol..

[14] Cortaga C.Q., Cordez B.W.P., Dacones L.S., Balendres M.A.O., De la Cueva F.M. (2023). Mutations associated with fungicide resistance in *Colletotrichum* species. Phytoparasitica.

[15] Leandero-Valenzuela N., Lara-Viveros F.M., Andrade-Hoyos P., Aguilar-Pérez L.A., Aguado-Rodríguez G.J. (2016). Alternativas para el control de *Colletotrichum* spp.. Rev. Mexicana Cienc. Agric..

[16] Chávez-Ramírez B., Kerber-Díaz J.C., Acoltzi-Conde M.C., Ibarra J.A., Vásquez-Murrieta M.S., Estrada-de los Santos P. (2020). Inhibition of *Rhizoctonia solani* RhCh-14 and *Pythium ultimum* PyFr-14 by *Paenibacillus polymyxa* NMA1017 and *Burkholderia cenocepacia* CACua-24: A proposal for biocontrol of phytopathogenic fungi. Microbiol. Res..

[17] Chávez-Ramírez B., Rodríguez-Velázquez N.D., Mondragón-Talonia C.M., Avendaño-Arrazate C.H., Martínez-Bolaños M., Vásquez-Murrieta M.S., Estrada de los Santos P. (2021). *Paenibacillus polymyxa* NMA1017 as a potential biocontrol agent of *Phytophthora tropicalis*, causal agent of cacao black pod rot in Chiapas, Mexico. Antonie Leeuwenhoek.

[18] Gómez-de la Cruz I., Chávez-Ramírez B., Avendaño-Arrazate C.H., Morales-García Y.E., Muñoz-Rojas J., Estrada-de los Santos P. (2023). Optimization of *Paenibacillus* sp. NMA1017 application as a biocontrol agent for *Phytophthora tropicalis* and *Moniliophthora roreri* in cacao-growing fields in Chiapas, Mexico. Plants.

[19] Gómez-de la Cruz I., Martínez-Bolaños M., Chávez-Ramírez B., Estrada-de los Santos P. (2024). Biocontrol of *Hemileia vastatrix*, the causal agent of coffee leaf rust, by *Paenibacillus* sp. NMA1017. Plant Dis..

[20] Martínez-Morales A., Hernández-Hernández L.U., Osorio-Osorio R., Alia-Tejacal I., López-Martínez V., Bautista-Baños S., Sánchez D.G. (2008). Incidencia y severidad de *Botryodiplodia theobromae* en frutos de zapote mamey en Jalpa de Méndez, Tabasco, México. UDO Ag..

[21] Castro-Pérez L.M., Saquero M.J., Beltrán-Herrera J.D. (2003). Caracterización morfológica y patogénica de *Colletotrichum* spp. como agente causal de la antracnosis en *Dioscorea* sp.. Rev. Colomb. Biotecnol..

[22] Allers T., Lichten M. (2000). A method for preparing genomic DNA that restrains branch migration of holliday junctions. Nucleic Acids Res..

[23] White T.J., Bruns T., Lee S., Taylor J., Innis M.A., Sninsky J.J., Gelfand D.H., White T.J. (1989). Amplification and direct sequencing of fungal ribosomal RNA genes for phylogenetics. PCR Protocols: A Guide to Methods and Applications.

[24] Guindon S., Dufayard J.F., Lefort V., Anisimova M., Hordijk W., Gascuel O. (2010). New algorithms, and methods to estimate maximum-likelihood phylogenies: Assessing the performance of PhyML 3.0. Syst. Biol..

[25] Tamura K., Stecher G., Kumar S. (2021). MEGA11: Molecular Evolutionary Genetics Analysis Version 11. Mol. Biol. Evol..

[26] Oliveira R., Bouhmidi K., Trapero A., Moral J. (2005). Caracterización morfológica y cultural de aislados de *Colletotrichum* spp. causantes de la antracnosis del olivo. Bol. Sanid. Veg. Plagas.

[27] Weir B.S., Johnston P.R., Damm U. (2012). The *Colletotrichum gloeosporioides* species complex. Stud. Mycol..

[28] Montero-Tavera V., Morales-García J.L., González-Chavira M.M., Anaya-López J.L., Corona-Torres T. (2010). Diversidad genética, patogénica y morfológica del hongo *Colletotrichum gloeosporioides* (Penz.) de Michoacán, México. Rev. Mexicana Cienc. Agric..

[29] Crisci J.V., Stuessy T.F. (1980). Determining primitive character states for phylogenetic reconstruction. Syst. Bot..

[30] Ezziyyani M., Sánchez C.P., Requena M.E., Rubio L., Castillo M.E.C. (2004). Biocontrol por *Streptomyces rochei*–Ziyani–, de la podredumbre del pimiento (*Capsicum annuum* L.) causada por *Phytophthora capsici*. Ann. Biol..

[31] Mohali-Castillo S.R., Stewart J.E. (2022). First report of *Colletotrichum siamense* associated with anthracnose on *Theobroma cacao* fruits in Venezuela. New Dis. Rep..

[32] Hernández-Gómez E., Hernández-Morales J., Avendaño-Arrazate H., López-Guillen G., Garrido-Ramírez R., Romero-Nápoles J., Romero-Nápoles J., Nava-Díaz C. (2015). Factores socieconómicos y parasitológicos que limitan la producción del cacao en Chiapas, México. Rev. Mex. Fitopatol..

[33] Gobierno del Estado de Chiapas (2010). Programa Regional 513 de Desarrollo Región X Soconusco. Secretaría de Desarrollo y Obras Públicas. https://www.haciendachiapas.gob.mx/planeacion/Informacion/Desarrollo-Regional/prog-regionales/SOCONUSCO.pdf.

[34] Mohanan R.C., Kaveriappa K.M., Nambiar K.K.N. (1989). Epidemiological studies of *Colletotrichum gloeosporioides* disease of cocoa. Ann. Appl. Biol..

[35] Crouch J.A., Beirn L.A., Cortese L.M., Bonos S.A., Clarke B.B. (2009). Anthracnose disease of switchgrass caused by the novel fungal species *Colletotrichum* navitas. Mycol. Res..

[36] Hyde K.D., Cai L., Cannon P.F., Crouch J.A., Crous P.W., Damm U., Goodwin P.H., Chen H., Johnston P.R., Jones E.B.G. (2009). *Colletotrichum* names in current use. Fungal Divers..

[37] Torres-Calzada C., Tapia-Tussell R., Higuera-Ciapara I., Huchin-Poot E., Martin-Mex R., Nexticapan-Garcez A., Perez-Brito D. (2018). Characterization of *Colletotrichum truncatum* from papaya, pepper and physic nut based on phylogeny, morphology and pathogenicity. Plant Pathol..

[38] Penet L., Guyader S., Petro D., Salles M., Bussière F. (2014). Direct splash dispersal prevails over indirect and subsequent spread during rains in *Colletotrichum gloeosporioides* infecting yams. PLoS ONE.

[39] Damm U., Cannon P.F., Woudenberg J.H.C., Crous P.W. (2012). The *Colletotrichum acutatum* species complex. Stud. Mycol..

[40] Mohamed A.I., Sundram S., Ramachandran V., Abu S.I. (2017). An in vitro investigation of Malaysian *Phytophthora palmivora* isolates and pathogenicity study on oil palm. J. Phytopathol..

[41] Manova V., Stoyanova Z., Rodeva R., Boycheva I., Korpelaien H., Vesterien E., Wirta H., Bonchev G. (2022). Morphological, pathological and genetic diversity of the *Colletotrichum* species, pathogenic on solanaceus vegetable crops in Burlgaria. J. Fungi.

[42] Yee M.F., Sariah M. (1983). Comparative morphology and characterization of *Colletotrichum* isolates occurring on cocoa in Malaysia. Pertanika J. Trop. Agric. Sci..

[43] Cannon P.F., Damm U., Johnston P.R., Weir B.S. (2012). *Colletotrichum* current status and future directions. Stud. Mycol..

[44] Lelwala R.V., Korhonen P.K., Young N.D., Scott J.B., Ades P.K., Gasser R.B., Taylor P.W. (2019). Comparative genome analysis indicates high evolutionary potential of pathogenicity genes in *Colletotrichum tanaceti*. PLoS ONE.

[45] Kim Y.S., Balaraju K., Jeon Y. (2016). Biological control of apple anthracnose by *Paenibacillus polymyxa* APEC128, an antagonistic *Rhizobacterium*. Plant Pathol. J..

[46] Kim Y.S., Balaraju K., Jeon Y. (2016). Effects of rhizobacteria *Paenibacillus polymyxa* APEC136 and *Bacillus subtilis* APEC170 on biocontrol of postharvest pathogens of apple fruits. J. Zhejiang Univ. Sci. B.

[47] Suryanto D., Wahyuni S., Siregar E.B.M., Munir E. (2014). Utilization of chitinolytic bacterial isolates to control anthracnose of cocoa leaf caused by *Colletotrichum gloeosporioides*. Afr. J. Biotechnol..

[48] Carrillo-Fasio J.A., García-Estrada R.S., Muy-Rangel M.D., Sañudo-Barajas A., Márquez-Zequera I., Allende-Molar R., de la Garza Ruiz Z., Vera M.P., Fentanes E.G. (2005). Control biológico de antracnosis *Colletotrichum gloeosporioides* (Penz.) Penz. y Sacc. y su efecto en la calidad postcosecha del mango (*Mangifera indica* L.) en Sinaloa, México. Rev. Mex. Fitopatol..

[49] Galindo E., Serrano-Carreón L., Gutiérrez C., Allende R., Balderas K., Patiño M., Trejo M., Wong M.A., Rayo E., Isauro D. (2013). The challenges of introducing a new biofungicide to the market: A case study. Electron. J. Biotechnol..

[50] Shi X., Wang S., Duan X., Wang Y., Liu F., Laborda P. (2021). Biocontrol strategies for the management of *Colletotrichum* species in postharvest fruits. Crop Prot..

[51] Lee B., Chen P., Chen C. (2024). Suppression of strawberry anthracnose by *Paenibacillus polymyxa* TP3 in situ and from a distance. Plant Dis..

[52] Sinuco-León D.C., Coconubo-Guio L.C., Castellanos-Hernández L. (2020). Fungicidal activity of volatile organic compounds from *Paenibacillus* bacteria against *Colletotrichum gloeosporioides*. Rev. Colomb. Quim..

