# Supplementary material for: Isolation and Biological Control of Colletotrichum sp. Causing Anthracnosis in Theobroma cacao L. in Chiapas, Mexico
Source: J Fungi (Basel). 2025 Apr 15;11(4):312. doi: 10.3390/jof11040312 (PMC12028460; doi:10.3390/jof11040312)
Supplement: Supplementary file 1 [file jof-11-00312-s001.zip › jof-3520823-supplementary.pdf]

Supplementary material

Isolation and biological control of *Colletotrichum* sp. causing anthracnosis in *Theobroma cacao* in Chiapas, México

Nadia Denisse Rodríguez Velázquez[1,2], Irene Gómez-de la Cruz[1,2], Guillermo López-Guillén[3], Belén Chávez-Ramírez[2]*, Paulina Estrada-de los Santos[1]*

[1]Instituto Politécnico Nacional, Escuela Nacional de Ciencias Biológicas, Laboratorio de Biotecnología Microbiana. Prol. Carpio y Plan de Ayala s/n. Col. Santo Tomás, Alcaldía Miguel Hidalgo, Ciudad de México. C.P. 11340.

[2]Instituto Politécnico Nacional, Escuela Nacional de Ciencias Biológicas, Laboratorio de Fitopatología. Prol. Carpio y Plan de Ayala s/n. Col. Santo Tomás, Alcaldía Miguel Hidalgo, Ciudad de México. C.P. 11340.

[3]Instituto Nacional de Investigaciones Forestales, Agrícolas y Pecuarias, Campo Experimental Rosario Izapa. Laboratorio de Sanidad Vegetal. Km 18, carretera Tapachula-Cacahoatán, Tuxtla Chico, Chiapas.

*Corresponding authors:

Belén Chávez-Ramírez, email: bchavezra@ipn.mx

Paulina Estrada-de los Santos, email: pestradadelossantos@gmail.com

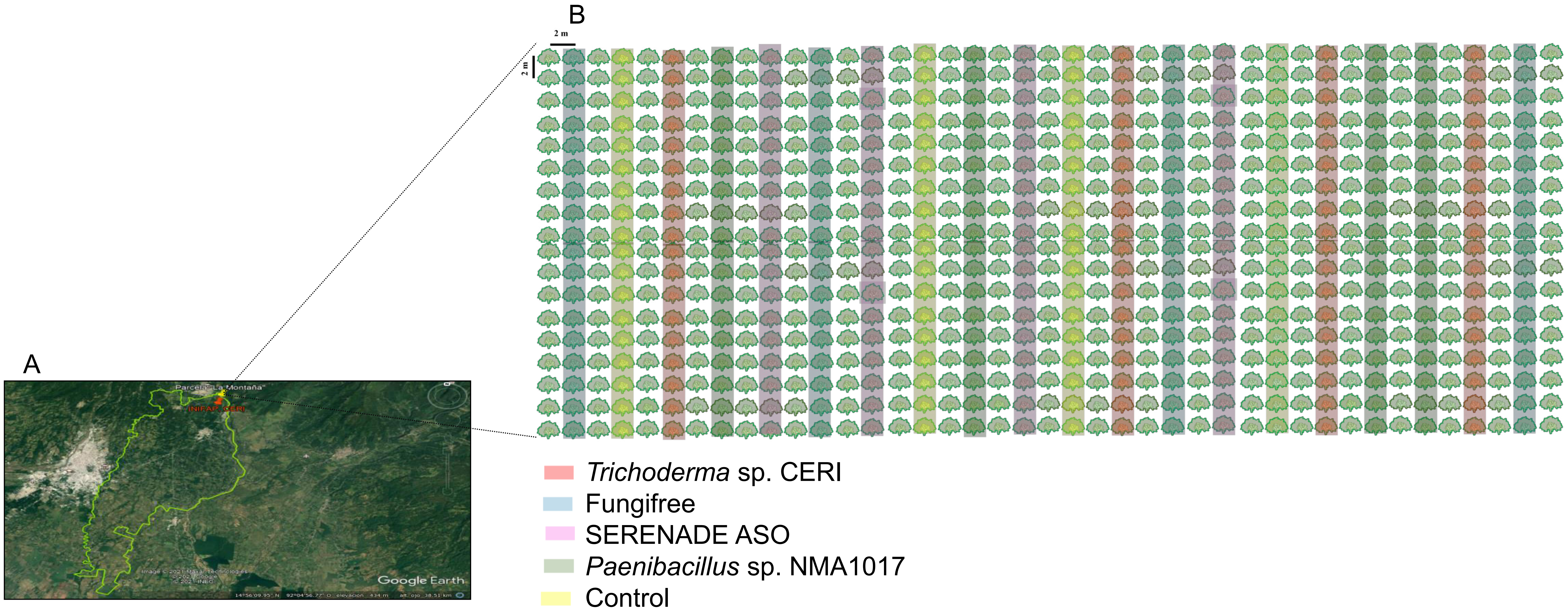

Figure S1. Location in Chiapas, Mexico (A) and experimental design (B) for applying *Paenibacillus* sp. NMA1017 to cacao trees for biocontrol of anthracnose. Each background color corresponds to different treatments. The white background color corresponds to a barrier between treatments.

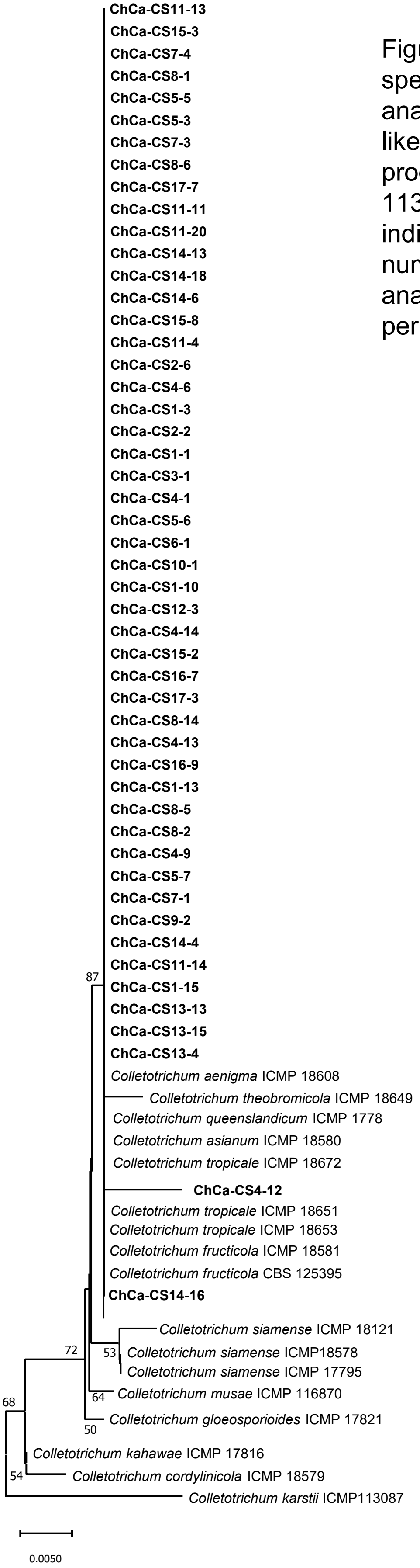

Figure S2. Phylogenetic analysis of *Colletotrichum* species using the ITS region sequence. The analysis was performed with the maximum likelihood method and the GTR+I model with the program PhyML v 3.0. *Colletotrichum karstii* ICMP 113087 was used as an outgroup. In bold letters are indicated the strains analyzed in this study. The numbers in the tree correspond to the bootstrap analysis (1000). Bar, 0.0050 nucleotide substitutions per position.

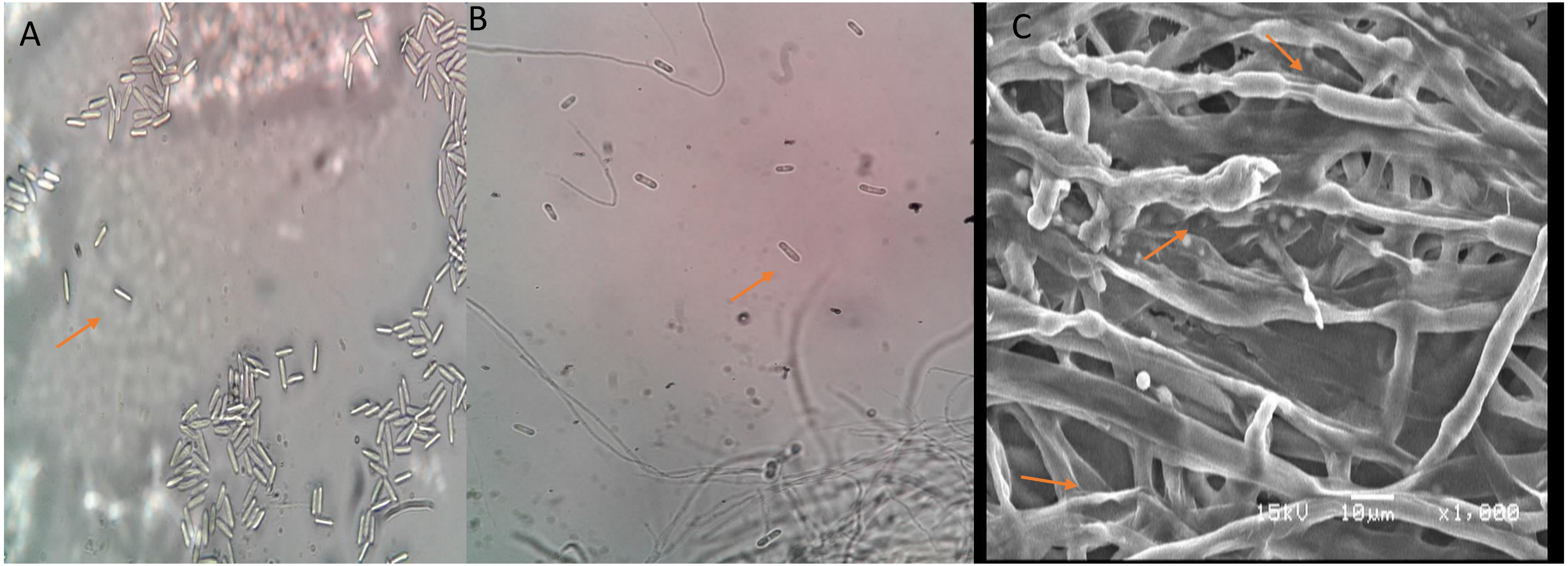

Figure S3. Effect of *Paenibacillus* sp. NMA1017 on *Colletricuhum* sp. in microcultures test. A, optical observation of the control (40×), the image shows many conidia and the arrow indicates typical conidia. B, optical observation of the effect of strain NMA1017 vs. *Colletotrichum* sp. (40×), the arrow points out a few conidia. C, scanning electron microscopy of the effect of strain NMA1017 vs *Colletotrichum* sp, the arrow specify damage to hypae.

Table S1. Morphological and physiological features of *Colletotrichum* spp. isolated from diseased cacao pods in Chiapas, Mexico.

MORPHOLOGICAL CHARACTERIZATION		PHYSIOLOGICAL CHARACTERIZATION	
**Colonial texture**	**% of isolates**	**Growth rate (mm/day)**	**% of isolates**
Powdery	51.79	6-6.9	8.93
Velvety	10.71	7-7.9	16.07
Cottony	37.50	8-8.9	33.93
**Color of the front side**		9-9.9	19.64
Gray	78	10-19.9	21.43
White	8.50	**Growth at different temperatures**	
Brown-black	4.20	28°C	100
Cream	2.10	30°C	100
**Color or the reverse side**		37°C	30
Pink	6.90	45°C	0
Gray	13.90	**Growth in different culture media**	
Black	62.70	PDA	100
Orange-pink	6.90	V8 agar	100
Brown	9.30	Cornmeal agar	100
**Presence of pycnidia**	78.57		
**Presence of concentric halos**	19.64		
**Cylindrical conidia**	85.8		
**Ovoid conidia**	14.2		
**Hyaline and septate mycelium**	100		

Table S2. Size of representative strains of *Colletotrichum* spp.

Strain	Size (µm)
ChCa-CS1-3	4.96 – 13.92 x 2.69 – 8.29 (average: 11.92 x 3.66)
ChCa-CS1-4	4.82 – 15.75 x 2.59 – 7.81 (average: 13.55 x 3.68)
ChCa-CS1-13	4.18 – 12.82 x 2.2 – 6.54 (average: 10.16 x 3.12)
ChCa-CS1-19	5.08 – 14 x 3.01 – 7.82 (average: 11.29 x 4.05)
ChCa-CS1-20	4.71 – 12.66 x 1.77 – 7.74 (average: 10.63 x 3.09)
ChCa-CS1-30	6.17 – 15.36 x 2.24 – 7.75 (average: 11.20 x 3.74)
ChCa-CS4-1	3.73 – 12.13 x 2.36 – 6.1 (average: 10.07 x 2.87)
ChCa-CS11-17	5.52 – 13.9 x 2.3 – 7.54 (average: 10.80 x 3.46)
ChCa-CS15-3	5.38 – 19 x 2.69 – 9.06 (average: 12.29 x 3.96)
